# Alkali Activation of Natural Calcium Bentonite for Foundry Applications: Structural, Physicochemical, and Technological Characterization

**DOI:** 10.3390/ma19132822

**Published:** 2026-07-02

**Authors:** Dragan Radulović, Jovica Stojanović, Marija Marković, Dejan Todorović, Vladimir Jovanović, Anja Terzić

**Affiliations:** 1 Institute for Technology of Nuclear and Other Mineral Raw Materials, Franchet d’Esperey 86, 11000 Belgrade, Serbia; d.radulovic@itnms.ac.rs (D.R.); j.stojanovic@itnms.ac.rs (J.S.); m.markovic@itnms.ac.rs (M.M.); d.todorovic@itnms.ac.rs (D.T.); v.jovanovic@itnms.ac.rs (V.J.); 2 Institute for Testing of Materials, Bulevar Vojvode Mišića 43, 11000 Belgrade, Serbia

**Keywords:** alkali activation, bentonite, montmorillonite, sodium activation, foundry binder, green sand molds, cation exchange capacity

## Abstract

The technological performance of bentonite in foundry applications is strongly influenced by the nature of its exchangeable interlayer cations, with sodium bentonites generally exhibiting superior swelling, plasticity, and bonding properties compared with calcium bentonites. Given the limited availability of natural sodium bentonite, upgrading abundant calcium-rich bentonite resources holds significant industrial interest. In this study, a natural Ca-rich bentonite from the Bijelo Polje deposit (Bar, Montenegro) was upgraded by alkali activation using Na_2_CO_3_ and evaluated as a binder for green sand foundry molds. The raw bentonite was characterized by physicochemical, mineralogical, and structural analyses, confirming its Ca-type character and suitability for sodium activation. Activation was performed using 2–6 wt.% Na_2_CO_3_, with the optimum treatment achieved at 5 wt.% Na_2_CO_3_. The activated bentonite was subsequently characterized using structural, textural, thermal, and physicochemical methods. Alkali activation significantly improved the key technological properties of the material, increasing the free swelling capacity from 7 to 20 cm^3^, the specific surface area from 27.4 to 45.8 m^2^ g^−1^, the cation exchange capacity from 74.6 to 89.5 meq/100 g, and the plasticity index from 79.6% to 193.4%. XRD, ATR–FTIR, and thermal analyses confirmed successful sodium activation while preserving the fundamental montmorillonite structure. Evaluation of foundry-relevant properties, including refractoriness, methylene blue adsorption, gas permeability, thermal stability, and bonding strength, demonstrated that the activated bentonite satisfies the technological requirements for green sand molding of both ferrous and non-ferrous alloys. These findings demonstrate that Na_2_CO_3_ activation is an effective and resource-efficient approach for converting natural Ca-rich bentonite into a high-performance foundry binder.

## 1. Introduction

Clay minerals constitute one of the most abundant groups of non-metallic mineral resources in the Earth’s crust and occur in a wide range of geological environments and structural forms [1]. Among these, bentonite is a naturally occurring clay material predominantly composed of minerals belonging to the smectite group. It is mainly formed through the alteration of volcanic ash and tuff deposits, but it can also come from the weathering of some igneous and sedimentary rocks under certain geological conditions [2]. Owing to its unique layered structure and distinctive physicochemical properties, bentonite has attracted considerable industrial interest. These properties include fine particle size, high specific surface area, elevated cation exchange capacity, pronounced swelling behavior, and strong adsorption affinity [3]. Such characteristics underpin its widespread industrial use across diverse sectors [4,5,6]. Bentonite is particularly important as a binder in green sand foundry systems, where its swelling and rheological behavior directly determine mold performance [7,8,9]. Other important applications of bentonite are petroleum drilling fluids [10], iron ore pelletization [11], and construction materials [12,13]. Driven by technological progress and growing environmental challenges, the utilization of bentonite is increasingly extending into emerging applications, including energy storage composites [14,15,16], wastewater treatment [17], and environmental remediation technologies [18]. Conventional applications, particularly in foundry industries [7], continue to dominate its industrial consumption. This dual relevance underscores bentonite’s strategic importance as a versatile mineral resource across both traditional and advanced technological sectors.

Bentonite properties are primarily determined by montmorillonite and its exchangeable interlayer cations [19,20,21]. The composition of these cations strongly influences swelling behavior, cation exchange capacity, and technological performance [22,23]. In addition, natural bentonites commonly contain accompanying minerals such as quartz, feldspars, calcite, and cristobalite [24,25]. Bentonite is extensively used as a binder in green sand molding systems, where hydrated montmorillonite platelets form cohesive clay–water networks around silica sand grains, providing the mechanical strength required for mold stability during casting [26,27]. The effectiveness of this binding mechanism depends strongly on interlayer hydration and exchangeable cation chemistry. Sodium bentonites generally exhibit higher swelling capacity, greater water absorption, and superior bonding performance than calcium bentonites due to enhanced interlayer hydration and clay dispersion [28]. Because high-quality natural sodium bentonite deposits are relatively scarce, calcium-rich bentonites are commonly upgraded by alkali activation using sodium carbonate, which promotes exchange of Ca^2+^ by Na^+^ within the interlayer space [29,30]. This process enhances swelling, plasticity, and binder performance and is therefore widely applied in industrial foundry practice [31]. In addition to swelling and bonding properties, thermal stability is an important performance criterion for foundry bentonites, as exposure to elevated temperatures during casting may induce dehydration and structural changes that influence mold integrity and casting quality [32,33].

Recent innovations in bentonite modification have focused on tailoring its physicochemical properties through surfactant, polymer, and nanostructured additives to expand its functionality beyond conventional foundry applications. Surfactant modification, particularly through quaternary ammonium compounds, converts hydrophilic bentonite into organophilic materials with increased interlayer spacing and enhanced affinity for organic species. These modified clays have shown considerable potential in environmental remediation and non-aqueous systems, including pollutant adsorption and oil-based drilling fluids, where improved dispersion and rheological control are required [34,35,36,37,38,39]. Polymer modification has further enabled improvements in mechanical stability, impermeability, and chemical resistance, supporting applications in barrier engineering and sealing systems [40,41]. More recently, nanostructured additives, including metal oxides and carbon-based nanomaterials, have emerged as advanced modifiers capable of enhancing thermal stability, mechanical strength, and surface reactivity, thereby enabling high-performance multifunctional bentonite composites [42,43].

Despite significant advances in surfactant, polymer, and nanostructured bentonite modification, alkali activation remains the most industrially relevant and economically viable approach for large-scale upgrading in foundry practice. Unlike advanced surface-functionalization strategies, which are typically designed for specialized applications, alkali activation directly modifies exchangeable interlayer cation composition and hydration behavior, thereby enhancing swelling capacity, clay dispersion, and binder performance. These improvements are essential in green sand molding systems, where stable clay–water–sand interactions govern mold strength, permeability, and dimensional stability during casting. However, most previous studies have focused primarily on isolated physicochemical parameters, such as swelling index and cation exchange capacity, without establishing direct correlations between mineralogical transformations and foundry-relevant engineering properties, particularly green strength, permeability, and thermal stability. Consequently, a comprehensive understanding of the relationship between alkali-induced structural modifications and functional foundry performance remains limited. Several critical knowledge gaps nevertheless remain regarding the optimization of alkali-activated bentonite for foundry applications. First, the relationship between interlayer cation exchange and functional foundry performance has not been sufficiently clarified. Second, despite their widespread availability, Ca-rich bentonites remain insufficiently explored as precursor materials for producing higher-performance Na-type binders. Third, integrated studies combining structural characterization with direct evaluation of foundry performance are still limited. Addressing these challenges is essential for developing resource-efficient activation strategies and improving the utilization of abundant low-grade bentonite resources in green sand molding systems. Although sodium activation of calcium bentonites has been extensively investigated, the response of individual bentonite deposits to activation depends strongly on their mineralogical composition, exchangeable cation distribution, and associated non-clay mineral assemblages. Consequently, comprehensive evaluation of locally available bentonite resources remains essential for assessing their suitability as alternatives to commercial sodium bentonites.

The novelty of the present work lies in four principal aspects. First, an integrated structure–property–performance approach was employed to systematically correlate exchangeable cation redistribution, mineralogical and structural modifications (XRD, ATR–FTIR, and DSC/TG), textural evolution (BET/BJH analyses), and foundry-relevant technological performance. While previous studies have frequently focused on isolated physicochemical characteristics such as swelling capacity, cation exchange capacity, or mineralogical changes following activation, comprehensive investigations linking these parameters to engineering performance remain comparatively limited. Second, the study provides the first comprehensive evaluation of sodium activation of bentonite from the Bijelo Polje deposit (Montenegro) for foundry applications. Because the efficiency of alkali activation is highly dependent on the mineralogical composition and exchangeable cation population of a given deposit, deposit-specific investigations remain important for assessing industrial applicability and resource utilization potential. Third, the work establishes direct structure–property–performance relationships by linking Na^+^ enrichment and interlayer cation exchange to changes in basal spacing, specific surface area, hydration behavior, swelling capacity, and ultimately to improvements in foundry-relevant properties, including green compressive strength, green shear strength, wet tensile strength, thermal stability, and gas permeability. Such quantitative correlations are less frequently reported than studies focused solely on physicochemical characterization. Finally, the industrial relevance of the activated material was assessed through comparison with performance criteria and literature data for commercial sodium bentonites used in green sand molding systems. This evaluation demonstrates the potential of the investigated bentonite resource as a locally available alternative to imported sodium bentonites and contributes to the development of resource-efficient foundry binders.

Accordingly, the objective of the present study was not only to investigate the effect of Na_2_CO_3_ activation on a locally sourced Ca-rich bentonite, but also to establish quantitative structure–property–performance relationships linking interlayer cation exchange, mineralogical transformations, textural evolution, and foundry-relevant technological properties. To achieve this objective, the raw and activated bentonites were comprehensively characterized using chemical analysis, exchangeable cation determination, X-ray diffraction (XRD), ATR–FTIR spectroscopy, thermal analysis (DSC/TG), and nitrogen adsorption–desorption measurements. The effectiveness of activation was further evaluated through swelling, hydration, adsorption, and foundry-related technological tests, and the resulting properties were compared with those of commercial sodium bentonites reported in the literature.

## 2. Materials and Methods

### 2.1. Raw Material Sampling and Preparation

Raw bentonite sample (RB) was collected from the open-pit exploration area of the “Bijelo Polje” bentonite deposit near Bar, Montenegro. Sampling was performed across lithologically distinct exposed sections to ensure representativeness of the mineralogical variability of the deposit. Individual subsamples were selected based on visually distinguishable variations in color and texture and subsequently homogenized and reduced by quartering to obtain a representative bulk sample for laboratory investigation.

The representative raw bentonite sample was air-dried, manually disaggregated, and homogenized prior to further preparation and characterization. Moisture content was determined gravimetrically by drying at 105 °C to constant mass in accordance with standard laboratory procedures.

For subsequent analyses, the raw bentonite was dried, crushed, and milled using a Retsch ZM1 centrifugal mill (Haan, Germany) to obtain a powder fraction smaller than 0.074 mm. The prepared material was homogenized and designated as the representative raw bentonite sample (RB). This sample was subsequently used for all physicochemical, chemical, mineralogical, structural, thermal, textural, and technological characterizations, as well as for the alkali-activation experiments.

### 2.2. Alkali Activation Procedure

Alkali activation was performed by mixing dried raw bentonite with sodium carbonate (Na_2_CO_3_) and distilled water at a water-to-solid ratio of 0.35:1 (*w*/*w*) until a homogeneous plastic mass was obtained. The Na_2_CO_3_ dosage was varied between 2 and 6 wt.% relative to the dry bentonite mass to determine the optimal activation level. Mixing was carried out for 15 min using a laboratory mixer.

The prepared plastic mass was aged for 24 h at room temperature (23 ± 2 °C) in sealed polyethylene containers to promote ion exchange and moisture equilibration. An aging period of 24 h was selected based on previous studies and industrial sodium-activation practice, which indicate that this duration is generally sufficient to promote interlayer cation exchange, moisture equilibration, and stabilization of the activated bentonite structure prior to further processing. The selected 24 h aging period ensured adequate ion exchange before extrusion and is consistent with activation times commonly reported for sodium activation of calcium bentonites.

The aged mixture was subsequently extruded three consecutive times through a perforated die using a laboratory extruder to ensure homogeneous distribution of the activating agent and effective disruption of clay agglomerates, with an approximate residence time of 3 min between successive passes.

The extruded material was pelletized and dried at 60 °C for 24 h to a residual moisture content of approximately 10 wt.%. No additional post-drying curing treatment was applied. The dried pellets were crushed and micronized to a particle size below 0.074 mm using a Retsch ZM1 centrifugal mill.

Activation efficiency was assessed by free swelling capacity measurements performed in triplicate, and the sample exhibiting the highest mean swelling value was selected as the optimally activated bentonite (AB).

### 2.3. Physicochemical and Chemical Characterization

The density of the investigated samples was determined using the pycnometer method. Free swelling capacity was measured in distilled water (2 g/100 mL) according to ASTM International ASTM D5890 [44].

The pH of the bentonite suspension was measured using a calibrated pH meter in a 1:10 (*w*/*v*) bentonite-to-distilled-water suspension after equilibration at room temperature.

Methylene blue adsorption capacity (MB) and cation exchange capacity (CEC) were determined by the methylene blue adsorption method, while the type and concentration of exchangeable cations were determined using the ammonium acetate method.

Chemical composition, including the identification and quantification of major oxides, was determined by atomic absorption spectroscopy (AAS) using a PinAAcle 900 instrument (PerkinElmer, Waltham, MA, USA).

### 2.4. Mineralogical and Structural Characterization

Mineralogical composition was determined using a SmartLab X-ray powder diffractometer (Rigaku Corporation, Tokyo, Japan) operating in Bragg–Brentano parafocusing geometry (CBO optics, BB slit). A ceramic copper X-ray tube with long fine focus served as the radiation source, operating at 40 kV and 30 mA. Diffraction patterns were recorded over the angular range of 4–65° 2θ using a D/teX Ultra 250 strip detector (Rigaku Corporation, Tokyo, Japan), with a step size of 0.01° and a collection speed of 0.5 min/° 2θ. Phase identification was performed using the International Centre for Diffraction Data PDF-4+ database, and diffractogram processing was carried out using SmartLab Studio II software, version 4.6.

Structural analysis was performed by attenuated total reflectance Fourier-transform infrared (ATR-FTIR) spectroscopy using a Thermo Fisher Scientific Nicolet iS50 spectrometer (Waltham, MA, USA) equipped with a single-bounce diamond ATR crystal. Spectra were recorded over the range of 4000–400 cm^−1^ at a resolution of 2 cm^−1^, with 64 sample scans and 64 background scans accumulated for each measurement. Post-acquisition processing included automatic baseline correction and atmospheric suppression. Spectral acquisition and processing were performed using OMNIC software (Version 8.0, Thermo Fisher Scientific, Waltham, MA, USA).

### 2.5. Thermal Analysis

Thermal behavior was investigated by simultaneous differential scanning calorimetry and thermogravimetric analysis (DSC/TG) using a NETZSCH STA 449 F5 Jupiter thermal analyzer (Selb, Germany). Approximately 20 mg of sample was placed in Pt/Rh crucibles and heated from 20 to 1000 °C at a heating rate of 10 °C min^−1^ under a static synthetic air atmosphere. The resulting thermograms were evaluated to identify dehydration, dehydroxylation, and thermally induced mineralogical transformations in the raw and alkali-activated bentonite samples. Thermogram acquisition and analysis were performed using Proteus software (Version 8.0, NETZSCH, Selb, Germany).

### 2.6. Surface Area and Porosity Analysis

Specific surface area (SBET) and pore characteristics were determined using a Micromeritics ASAP 2020 analyzer (Unterschleißheim, Germany). Prior to analysis, samples were degassed under vacuum at 120 °C for 12 h. Nitrogen adsorption–desorption isotherms were recorded at 77 K, and the Brunauer–Emmett–Teller (BET) method was applied to determine the specific surface area. Total pore volume, average pore diameter, and pore size distribution were calculated from the desorption branch using the Barrett–Joyner–Halenda (BJH) method. Data processing and pore structure calculations were performed using MicroActive software (version 6.0, Micromeritics Instrument Corporation, Norcross, GA, USA).

### 2.7. Technological Properties

The liquid limit (WLL) and plastic limit (WPL) were determined using the Casagrande cup and hand-rolling methods in accordance with ASTM D4318 [45] and ISO 17892 [46]. The plasticity index (PI) was calculated as the difference between WLL and WPL. Water absorption capacity was determined using the Enslin–Neff method (DIN 18132) [47]. Refractoriness was evaluated as pyrometric cone equivalent (PCE) according to ASTM C24 [48] and ISO 528 [49].

### 2.8. Foundry Performance Evaluation

The alkali-activated bentonite sample (AB) was evaluated for its applicability as a binder in green sand molding systems using Georg Fischer foundry laboratory equipment and established testing procedures. Test specimens were prepared using quartz sand KPL 026 with a median grain size of d50 = 0.26 mm and a grain uniformity coefficient of 91%, with the addition of 7 wt.% bentonite and 4 wt.% water relative to sand mass. The following foundry performance parameters were determined: green compressive strength, green shear strength, wet tensile strength, thermal stability at 500 °C, and gas permeability. For thermal stability evaluation, compacted test specimens were heated at 500 °C for 1 h, cooled to room temperature, and subsequently tested for residual compressive strength. Standard cylindrical specimens were prepared and tested. Green compressive strength, green shear strength, and wet tensile strength were determined on cylindrical green sand specimens prepared under controlled compaction conditions. Gas permeability was measured by determining the rate of airflow through the compacted specimen under a standardized pressure differential and is reported as the permeability number. These parameters were selected to evaluate the bonding efficiency, mold stability, and gas-venting characteristics of the bentonite-containing molding mixtures.

### 2.9. Statistical Analysis

All quantitative measurements related to physicochemical, technological, and foundry-performance properties were performed in triplicate unless otherwise stated. Results are reported as mean ± standard deviation (SD). Statistical differences between raw bentonite (RB) and alkali-activated bentonite (AB) were evaluated using Student’s *t*-test, with statistical significance accepted at *p* < 0.05. Instrumental analyses (XRD, ATR–FTIR, DSC/TG, and nitrogen adsorption–desorption measurements) were conducted on representative samples and are presented as individual measurements.

## 3. Results and Discussion

### 3.1. Characterization of the Raw Bentonite (RB)

The raw bentonite (RB) exhibited a moisture content of 23.78%, while its free swelling capacity was determined to be 7 cm^3^, indicating moderate swelling behavior characteristic of Ca-rich bentonites. The results of methylene blue (MB) adsorption, cation exchange capacity (CEC), and exchangeable cation distribution are presented in Table 1, while the chemical composition is summarized in Table 2.

The close agreement between the CEC value (Table 1) determined by methylene blue adsorption (74.60 meq/100 g) and the sum of exchangeable cations determined by the ammonium acetate method (76.19 meq/100 g) confirms the reliability of the analytical results and indicates that the majority of the exchangeable charge is associated with the smectite-rich fraction of the bentonite.

The chemical composition (Table 2) is characterized by a high SiO_2_ content (70.42 wt.%), accompanied by moderate Al_2_O_3_ content (12.53 wt.%) and relatively low concentrations of alkaline and alkaline-earth oxides. The elevated SiO_2_ content suggests the presence of significant quantities of non-clay siliceous phases, whose presence was subsequently confirmed by XRD (Section 3.1.1). The CaO content (2.47 wt.%) is consistent with the predominance of exchangeable calcium ions determined by the ammonium acetate method.

The predominance of Ca^2+^ ions, accounting for approximately 56.5% of the total exchangeable cation population, confirms that the investigated material can be classified as a calcium bentonite. The relatively low Na_2_O content further supports this classification. Using a typical CEC value of 105–110 meq/100 g reported for pure montmorillonite [19,20], the montmorillonite content of the investigated bentonite was estimated to be approximately 70 wt.%, indicating that smectite represents the dominant clay mineral phase.

The Ca-dominated exchange complex together with the relatively high montmorillonite content indicates substantial potential for sodium activation through interlayer cation exchange. Consequently, the investigated bentonite represents a suitable precursor material for evaluating the influence of sodium activation on mineralogical transformations, textural evolution, and foundry-relevant technological properties.

#### 3.1.1. Mineralogical Characterization of RB

**Figure 1:**
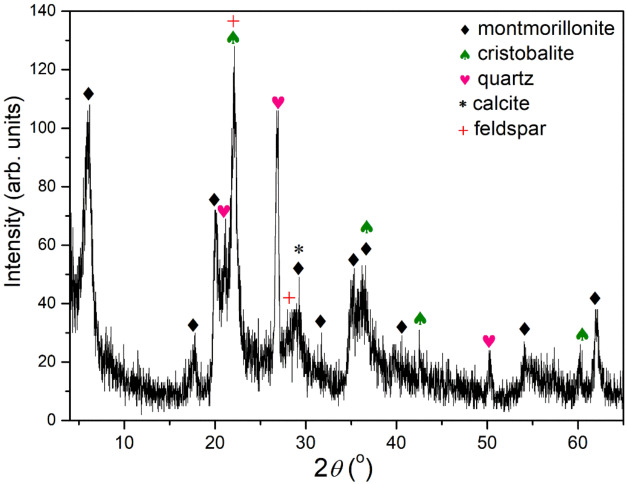
XRD pattern of RB sample.

The X-ray diffraction pattern of the RB sample is presented in Figure 1. The diffractogram confirms that montmorillonite is the dominant mineral phase, accompanied by quartz, cristobalite, feldspar-group minerals, and minor calcite. The predominance of montmorillonite is evidenced by the characteristic basal reflection (001) at 2θ = 5.98°, corresponding to a d001 spacing of 1.472 nm according to Bragg’s law. This basal spacing is typical of hydrated Ca-dominated montmorillonite and is consistent with the predominance of exchangeable Ca^2+^ ions determined by the ammonium acetate method (Table 1).

Besides montmorillonite, the XRD pattern revealed the presence of quartz, cristobalite, and minor calcite. The occurrence of quartz and cristobalite is consistent with the high SiO_2_ content determined by chemical analysis (AAS) in Table 2. Since the present XRD analysis was qualitative and no Rietveld refinement or other quantitative phase analysis was performed, the relative abundances of the identified mineral phases could not be reliably determined. The mentioned non-clay phases are commonly encountered in natural bentonites and may influence properties such as specific surface area, swelling behavior, and cation exchange capacity.

In addition to the basal reflection, montmorillonite is further identified by characteristic reflections at approximately 19.9°, 28.9°, 35.3°, 36.6°, 40.6°, 54.1°, and 61.9° 2θ, confirming that it represents the principal clay mineral phase in the investigated material. The estimated montmorillonite content derived from CEC measurements (~70 wt.%) is consistent with the predominance of montmorillonite indicated by the XRD pattern. Quartz is identified by its characteristic reflections at approximately 20.8°, 26.6°, and 50.1° 2θ, while cristobalite is recognized by reflections near 21.9°, 36.8°, 42.7°, and 60.3° 2θ. The occurrence of these silica phases is consistent with the elevated SiO_2_ content (70.42 wt.%) determined by chemical analysis (Table 2). Minor reflections attributable to feldspar-group minerals were observed at approximately 22.1° and 28.2° 2θ, whereas a weak reflection near 29.4° 2θ indicates the presence of a small amount of calcite. The presence of cristobalite is commonly associated with bentonites derived from altered volcanic ash and therefore provides additional evidence for the pyroclastic origin of the precursor material.

The coexistence of montmorillonite with quartz, cristobalite, feldspar-group minerals, and minor calcite is characteristic of bentonite deposits formed through the alteration of volcanic ash under variable diagenetic conditions. The relatively high abundance of montmorillonite, together with the predominance of exchangeable Ca^2+^ ions, confirms the classification of the investigated material as a Ca-rich bentonite and indicates favorable conditions for sodium activation through interlayer cation exchange. These mineralogical characteristics provide a baseline for evaluating structural modifications induced by alkali activation and their influence on the technological properties of the bentonite. No significant reflections attributable to other swelling clay minerals were observed, indicating that montmorillonite is the principal contributor to the measured cation exchange capacity, hydration behavior, and swelling properties of the RB sample.

#### 3.1.2. ATR–FTIR Analysis of RB

**Figure 2:**
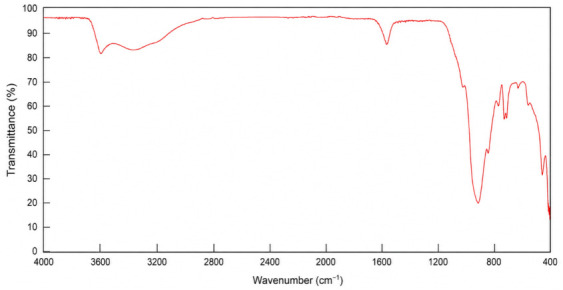
ATR–FTIR spectrum of RB sample.

The ATR–FTIR spectrum of the raw bentonite, recorded in the range of 4000–400 cm^−1^, is presented in Figure 2. The assignments of the principal ATR–FTIR absorption bands of the RB sample are summarized in Table 3.

The spectrum (Figure 2) exhibits absorption bands characteristic of smectite-group clay minerals, particularly montmorillonite. A broad band in the 3400–3450 cm^−1^ region is attributed to O–H stretching vibrations of adsorbed and interlayer water molecules. An absorption band centered at approximately 3620 cm^−1^ is assigned to the stretching vibrations of structural hydroxyl groups (Al–Al–OH) located within the octahedral sheets of the montmorillonite structure. The band observed near 1635 cm^−1^ corresponds to the H–O–H bending vibration of molecular water associated with adsorbed and interlayer water.

The most intense absorption band, located at approximately 1030 cm^−1^, is assigned to Si–O stretching vibrations within the tetrahedral silicate framework and represents a characteristic feature of montmorillonite. The pronounced intensity of the Si–O stretching band near 1030 cm^−1^ reflects contributions from both the montmorillonite framework and the silica-rich accessory phases identified by XRD analysis (Figure 1). Additional bands within the 1110–1000 cm^−1^ region are likewise attributed to Si–O stretching modes originating from the aluminosilicate framework. A weak band near 915 cm^−1^ is attributed to Al–Al–OH bending vibrations within the octahedral sheet.

Absorption bands in the 800–750 cm^−1^ region are associated with Si–O vibrations of crystalline silica phases. In particular, bands observed at approximately 792 and 772 cm^−1^ are characteristic of quartz and cristobalite, corroborating the presence of siliceous impurities identified by XRD. Furthermore, bands below approximately 550 cm^−1^ are attributed to Si–O–Al and Si–O–Si bending vibrations within the aluminosilicate framework. The ATR–FTIR results are in good agreement with the XRD findings and further confirm that montmorillonite is the dominant mineral phase in the investigated bentonite, accompanied by quartz and cristobalite as the principal non-clay constituents.

#### 3.1.3. Thermal Behavior of RB

**Figure 3:**
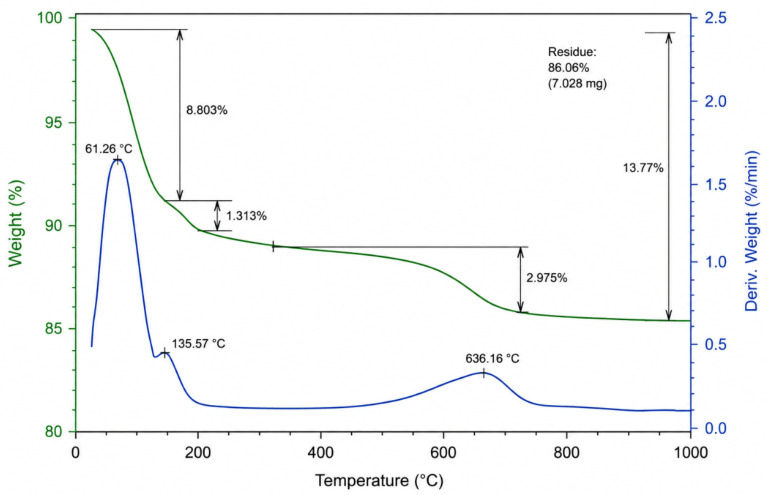
DSC/TG curves of RB sample.

The thermal behavior of the raw bentonite was investigated by simultaneous DSC/TG analysis, and the obtained thermograms are presented in Figure 3.

The thermograms reveal a multistage mass-loss process characteristic of smectite-rich bentonites. The first major thermal effect occurs below 150 °C and is characterized by a pronounced endothermic peak centered at approximately 61.3 °C. This process is accompanied by a mass loss of 8.80% and is attributed to the removal of physically adsorbed water and weakly bound interlayer water molecules. A second, less intense endothermic effect observed at approximately 136.6 °C is associated with the release of more strongly retained interlayer water, resulting in an additional mass loss of 1.31%.

A broad endothermic effect is observed at approximately 636 °C. This thermal effect is accompanied by a mass loss of 2.98% and corresponds to the dehydroxylation of the montmorillonite structure, involving the removal of structural hydroxyl groups from the octahedral sheets. The position of this peak is consistent with the dehydroxylation temperatures commonly reported for Ca-rich montmorillonitic bentonites.

The cumulative mass loss recorded up to 1000 °C was 13.77%, leaving a residual mass of 86.06%. The predominance of dehydration and dehydroxylation processes, together with the absence of significant additional thermal effects, suggests that the investigated bentonite contains only minor amounts of thermally reactive accessory phases. The dehydroxylation behavior is consistent with the presence of montmorillonite identified by XRD (Figure 1) and the structural hydroxyl groups detected by ATR–FTIR (Figure 2).

The mass loss attributed to dehydration (10.11%) is considerably greater than that associated with dehydroxylation (2.98%), reflecting the hydrated nature of the investigated bentonite and confirming the substantial contribution of adsorbed and interlayer water to its thermal behavior. This observation is consistent with the presence of hydrated Ca^2+^ ions inferred from the XRD (Figure 1) and exchangeable cation analyses (Table 1). The DSC/TG results confirm the smectite-rich character of the investigated bentonite and provide additional evidence for its Ca-dominated montmorillonitic composition.

#### 3.1.4. Textural Properties of RB

**Figure 4:**
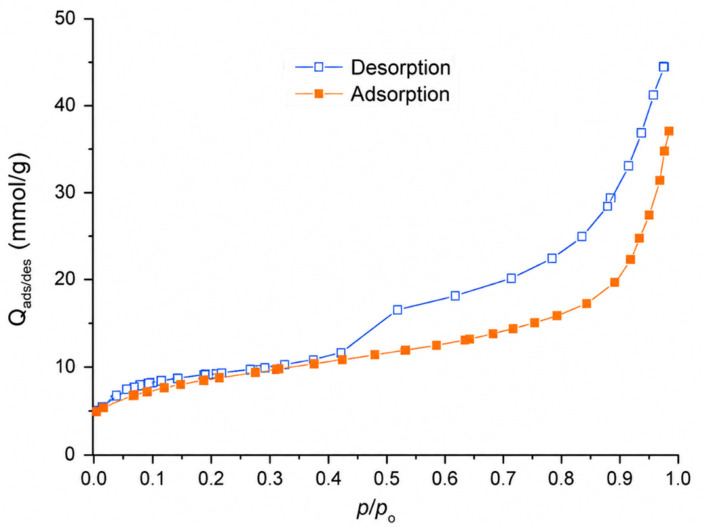
Nitrogen adsorption–desorption isotherm of RB sample.

**Figure 5:**
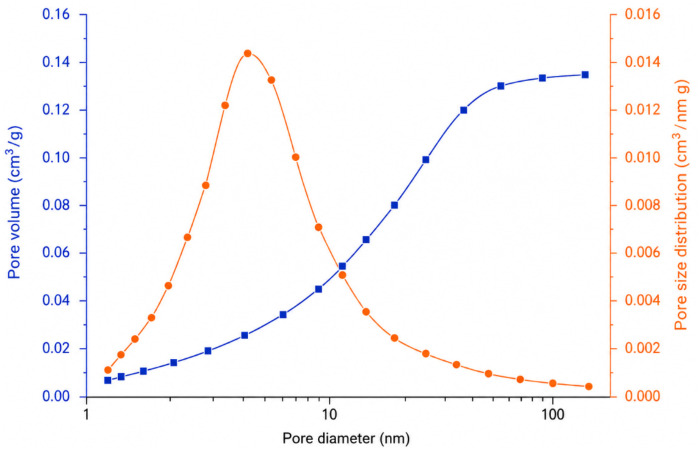
BJH pore size distribution and cumulative pore volume of RB sample.

The nitrogen adsorption–desorption isotherm and the corresponding BJH pore size distribution of the RB are presented in Figure 4 and Figure 5, respectively, while the calculated textural parameters are summarized in Table 4.

According to the IUPAC classification, the adsorption–desorption isotherm (Figure 4) corresponds to a type IV isotherm with a hysteresis loop, characteristic of mesoporous materials. This behavior is typical of smectite-rich aluminosilicates, where multilayer adsorption is followed by capillary condensation within mesopores.

As can be seen from Table 4, the RB sample exhibited a specific surface area (SBET) of 27.4 m^2^/g and a total pore volume of 0.0638 cm^3^/g. The pore system was dominated by mesopores, as indicated by the mesopore volume (0.0613 cm^3^/g), whereas the contribution of micropores was comparatively low (0.0023 cm^3^/g).

The specific surface area of the investigated bentonite (27.4 m^2^/g) is lower than values commonly reported for highly purified sodium montmorillonites. This behavior can be attributed to the mineralogical composition of the sample, which contains significant amounts of non-clay siliceous phases, including quartz and cristobalite, as confirmed by XRD analysis (Figure 1) and supported by the elevated SiO_2_ content (70.42 wt.%). These minerals possess substantially lower specific surface areas than smectite minerals and therefore reduce the overall BET surface area of the bulk sample. In addition, the predominance of Ca^2+^ as the exchangeable interlayer cation may contribute to a lower degree of layer expansion and reduced accessibility of internal surfaces compared with sodium-rich bentonites. The obtained SBET value is therefore consistent with the mineralogical and chemical characteristics of the investigated Ca-rich bentonite.

The BJH pore size distribution (Figure 5) exhibited a modal pore diameter of approximately 3.7 nm, whereas the average pore diameter was 8.5 nm, further confirming the mesoporous character of the investigated bentonite. The relatively broad pore size distribution reflects the heterogeneous arrangement of clay aggregates and associated interparticle voids typical of natural bentonites.

The predominance of mesoporosity is favorable for ion-exchange and adsorption processes because it facilitates the diffusion of water molecules and exchangeable cations within the clay aggregate structure. These textural characteristics are expected to influence ion-exchange processes during sodium activation by facilitating the diffusion of water molecules and exchangeable cations within the clay aggregate structure. Consequently, they may contribute to the technological performance of the activated bentonite.

#### 3.1.5. Technological Properties of RB

The Atterberg consistency limits determined for RB sample were as follows: liquid limit (WLL) = 118.70%, plastic limit (WPL) = 39.03%, and plasticity index (PI) = 79.63%. The water absorption capacity determined by the Enslin–Neff method was 171.39%. The high liquid limit and plasticity index indicate a pronounced capacity for water uptake and plastic deformation, which is characteristic of smectite-rich clay materials. These properties are primarily associated with the predominance of montmorillonite identified by XRD and supported by the relatively high cation exchange capacity of the sample.

Despite the high plasticity, the free swelling value of 7 cm^3^ and the predominance of exchangeable Ca^2+^ ions indicate that the investigated material is a Ca-rich bentonite. Compared with sodium bentonites, calcium bentonites generally exhibit lower swelling capacities because divalent interlayer cations promote stronger electrostatic interactions between adjacent clay layers, thereby restricting interlayer expansion.

The measured technological parameters demonstrate that the raw bentonite possesses favorable water-retention and plasticity characteristics. However, its moderate swelling capacity indicates that sodium activation may further improve hydration behavior, enhance interlayer expansion, and improve its performance in foundry applications.

#### 3.1.6. Integrated Discussion of Raw Bentonite Characteristics

The combined mineralogical, chemical, thermal, textural, and technological characterization results consistently indicate that raw bentonite is a Ca-rich bentonite dominated by montmorillonite. The predominance of exchangeable Ca^2+^ ions (56.5% of the total exchangeable cations), together with the relatively low Na_2_O content, confirms the calcium nature of the bentonite, while the measured CEC (74.60 meq/100 g) and estimated montmorillonite content (~70 wt.%) demonstrate the substantial contribution of the smectite phase to the overall physicochemical behavior of the material.

The mineralogical composition determined by XRD (Figure 1) is in excellent agreement with both the chemical (Table 2) and spectroscopic results (Figure 2). The high SiO_2_ content (70.42 wt.%) is explained by the presence of quartz and cristobalite identified in the diffractogram, while the characteristic FTIR bands at approximately 3620, 1635, and 1030 cm^−1^ confirm the presence of montmorillonite and associated interlayer water. The occurrence of cristobalite together with montmorillonite further supports the interpretation that the bentonite originated from the alteration of volcanic ash or pyroclastic precursor material.

The thermal behavior (Figure 3) of the sample is also consistent with the mineralogical composition. The dominant dehydration process below 150 °C reflects the presence of adsorbed and interlayer water associated with the hydrated Ca-montmorillonite structure, whereas the dehydroxylation peak at approximately 636 °C confirms the presence of structural hydroxyl groups within the octahedral sheets. The absence of significant additional thermal effects indicates that the accessory mineral phases occur only in minor amounts and do not substantially influence the thermal stability of the material.

The textural properties further support the observed mineralogical characteristics. Although the bentonite contains a high proportion of montmorillonite, the measured BET surface area (27.4 m^2^/g) is moderate compared with highly purified sodium montmorillonites. This behavior can be attributed to the significant proportion of non-clay siliceous minerals and the predominance of Ca^2+^ ions, which limit interlayer expansion and reduce the accessibility of internal surfaces. Nevertheless, the predominance of mesoporosity and the average pore diameter of 8.5 nm are favorable for mass transport, water penetration, and ion-exchange processes.

The technological properties reflect the combined influence of the mineralogical and textural characteristics. The high liquid limit, plasticity index, and water absorption capacity demonstrate substantial affinity toward water, which is typical of montmorillonitic clays. However, the moderate free swelling capacity is consistent with the Ca-dominated exchange complex, where divalent cations restrict interlayer expansion. Consequently, the investigated bentonite possesses significant potential for sodium activation, which is expected to increase swelling capacity, improve hydration behavior, enhance cation-exchange efficiency, and ultimately improve its suitability for foundry applications.

### 3.2. Characterization of Alkali-Activated Bentonite (AB)

The optimal Na_2_CO_3_ content for bentonite activation was determined by treating raw bentonite (RB) with 2–6 wt.% Na_2_CO_3_, as described in Section 2. The effectiveness of activation was evaluated based on the free swelling capacity of the resulting products. The maximum free swelling capacity (20 cm^3^) was obtained for the sample activated with 5 wt.% Na_2_CO_3_, compared with 7 cm^3^ for RB (Section 3.1). Therefore, the sample activated with 5 wt.% Na_2_CO_3_ was selected for further characterization and designated as AB. The characterization results of the activated bentonite (AB) are discussed below with emphasis on the physicochemical changes induced by sodium activation relative to the raw bentonite (RB).

As can be seen from Table 5, alkali activation increased the Na_2_O content from 0.51 to 2.22 wt.% while simultaneously reducing the CaO content from 2.47 to 1.85 wt.%, resulting in a Na_2_O/CaO ratio of approximately 1.2. The observed increase in Na_2_O content and decrease in CaO content indicate changes in the bulk chemical composition following treatment with Na_2_CO_3_. However, chemical analysis (AAS) alone cannot confirm the extent of cation exchange within the montmorillonite structure. Therefore, the effectiveness of sodium activation was further assessed using exchangeable cation analysis, cation exchange capacity measurements, free swelling tests, and structural characterization.

The physicochemical properties of RB and AB are summarized in Table 6. Alkali activation significantly enhanced the free swelling capacity, increasing it from 7 cm^3^ to 20 cm^3^. In addition, higher plasticity and water absorption capacity were observed for AB. These results are consistent with the conversion of Ca-bentonite to Na-bentonite, which is known to exhibit enhanced hydration and dispersion due to weaker interlayer interactions.

The more than twofold increase in liquid limit and the substantial increase in plasticity index indicate a pronounced enhancement of the hydration capacity and rheological behavior of the activated bentonite.

The exchangeable cation composition and cation exchange capacity (CEC) of RB and AB are presented in Table 7. Activation substantially decreased the Ca^2+^ content while increasing the Na^+^ content, resulting in an increase in CEC from 74.60 to 89.52 meq/100 g.

The proportion of exchangeable Na^+^ increased from approximately 19.5% of the total exchangeable cations in RB to approximately 68.8% in AB, whereas the contribution of exchangeable Ca^2+^ decreased from 56.5% to 17.3%, confirming the transition from a Ca-dominated to a Na-dominated exchange complex.

These shifts in exchangeable cation content provide more direct evidence of interlayer cation exchange than bulk chemical analysis alone, since CEC and exchangeable cation measurements reflect the actual ion-exchange capacity of the montmorillonite structure rather than overall elemental composition. Nonetheless, confirming the structural consequences of this exchange requires complementary evidence. Accordingly, the extent and impact of sodium activation were corroborated through free swelling tests and structural characterization, alongside the exchangeable cation and CEC data presented above.

The substantial increase in the Atterberg consistency limits following alkali activation reflects profound changes in the hydration behavior and surface chemistry of the bentonite. The liquid limit increased by approximately 106%, from 118.7% for RB to 244.6% for AB, while the plasticity index rose from 79.7% to 193.4%, indicating a markedly enhanced capacity of the activated material to retain water while maintaining plastic behavior. These changes can be primarily attributed to the replacement of exchangeable Ca^2+^ by Na^+^ within the montmorillonite interlayer structure, as evidenced by the exchangeable cation analysis (Table 7). Compared with divalent calcium ions, monovalent sodium ions exhibit lower charge density and weaker electrostatic interactions with the negatively charged clay layers. As a result, water molecules can more readily penetrate the interlayer space, leading to greater expansion of the diffuse double layer and enhanced hydration of the clay particles.

The increased hydration is further supported by the pronounced rise in free swelling capacity (7–20 cm^3^) and water absorption capacity (116.50–280.78%), which indicate a substantially greater affinity of the activated bentonite for water. In addition, the increase in the measured CEC from 74.60 to 89.52 meq/100 g suggests improved accessibility of exchange sites resulting from enhanced hydration and dispersion of the montmorillonite structure. The combined effects of enhanced interlayer expansion, diffuse double-layer development, and greater water retention directly contribute to the observed increases in liquid limit and plasticity index.

Similar behavior has been widely reported for sodium-activated bentonites, where replacement of exchangeable Ca^2+^ by Na^+^ promotes diffuse double-layer expansion, increased hydration, and enhanced swelling capacity [50,51]. The pronounced increases in Na^+^ content, free swelling capacity, water absorption, Atterberg consistency limits, methylene blue adsorption, and measured CEC collectively demonstrate the effectiveness of the sodium activation treatment. These results confirm the successful conversion of the investigated Ca-bentonite into a sodium-dominated bentonite with significantly improved hydration, rheological, and ion-exchange properties, which are highly desirable for foundry applications.

#### 3.2.1. XRD Analysis of AB and Comparison to RB

**Figure 6:**
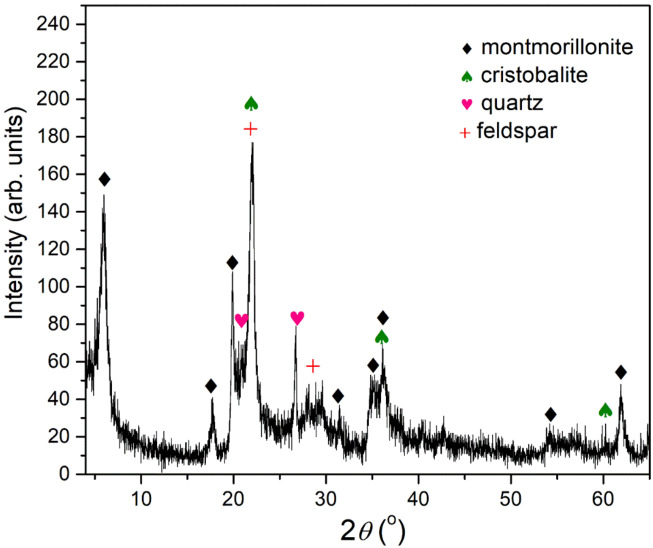
XRD patterns of alkali-activated bentonite (AB).

**Figure 7:**
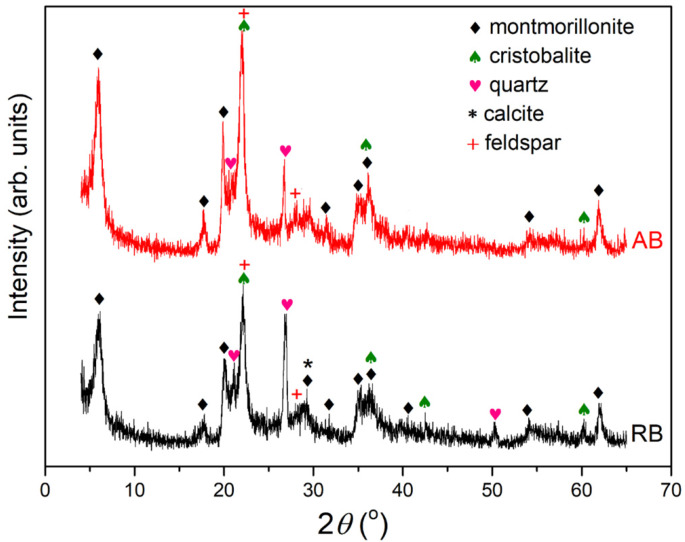
Comparison of XRD patterns of AB and RB samples.

The structural changes induced by alkali activation were further investigated by X-ray diffraction analysis, and the resulting diffractogram of sample AB is presented in Figure 6. The comparison of the obtained results is presented using stacked XRD diagrams of RB and AB samples is shown in Figure 7.

The XRD pattern of the AB sample (Figure 6) showed a shift in the principal montmorillonite (001) reflection from d001 = 1.47 nm (2θ = 5.98°) in RB to d001 = 1.23 nm (2θ = 7.16°). This shift toward higher diffraction angles indicates a reduction in basal spacing and reflects modifications of the interlayer environment resulting from sodium activation. The observed contraction is consistent with the replacement of hydrated exchangeable Ca^2+^ ions by Na^+^ ions and changes in the hydration state of the montmorillonite interlayers [25,26]. The reduced intensity of the (001) reflection may indicate partial modification of layer stacking and a decrease in long-range structural ordering following activation.

As can be observed in Figure 7, the weak diffraction peak at 2θ = 29.6°, attributed to calcite in the raw bentonite sample, was not observed after activation (sample AB). Previous studies have reported that during soda ash activation of Ca-rich bentonites, displaced Ca^2+^ ions may react with carbonate species to form amorphous or poorly crystalline calcium-containing phases, including CaCO_3_, Ca(OH)_2_, and Na_2_Ca(CO_3_)_2_·5H_2_O [26,50]. Therefore, the disappearance of the calcite reflection is likely associated with the transformation of calcium-containing phases during activation, possibly resulting in the formation of poorly crystalline carbonate-bearing products and/or reflections that overlap with those of montmorillonite and cristobalite.

The XRD results demonstrate that alkali activation primarily affected the interlayer chemistry of montmorillonite rather than its crystal structure. The observed shift in the basal reflection, together with the disappearance of calcite and the preservation of the major silicate phases, provides clear evidence of successful sodium activation. These structural modifications are consistent with the enhanced swelling capacity, increased cation exchange capacity, and improved technological performance observed for the activated bentonite, thereby confirming the effectiveness of the activation treatment.

#### 3.2.2. ATR–FTIR Analysis of AB and Comparison with RB

**Figure 8:**
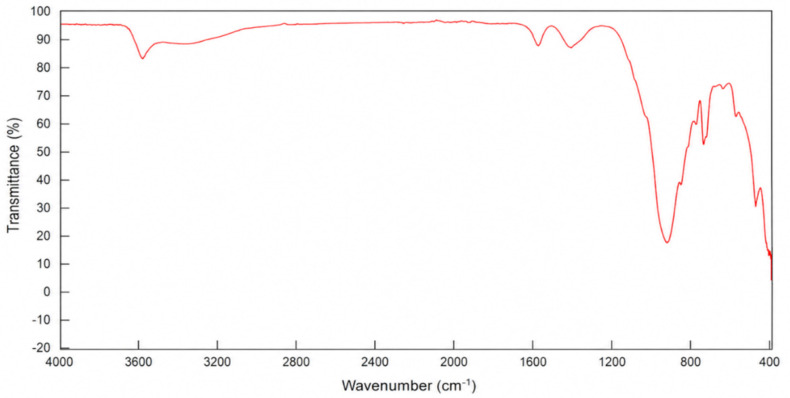
ATR–FTIR spectrum of AB sample.

**Figure 9:**
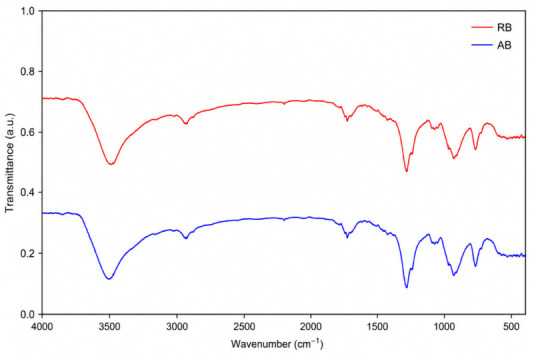
Comparison of ATR–FTIR spectra of AB and RB samples.

The ATR–FTIR spectrum of the AB sample is presented in Figure 8. For clarity and direct comparison of the spectral features before and after activation, the combined ATR–FTIR spectra of RB and AB are provided in Figure 9.

As it can be seen from Figure 8, the spectrum related to the AB sample exhibited characteristic absorption bands at approximately 3615, 1636, 1482, 985, 794, 510, 421, and 409 cm^−1^. Compared with the RB sample, as evidenced in Figure 9, a new absorption band appeared at 1482 cm^−1^, whereas the remaining bands associated with the montmorillonite framework showed no significant changes in position or intensity. These observations indicate that alkali activation primarily modified the interlayer chemistry while preserving the fundamental aluminosilicate structure of montmorillonite.

The absorption band at 1482 cm^−1^ (in Figure 8) is assigned to the ν_3_ asymmetric stretching vibration of carbonate groups [28,29], indicating the presence of carbonate-containing species formed during sodium activation. This observation is consistent with the use of Na_2_CO_3_ as the activating agent and supports the occurrence of cation-exchange reactions between Na^+^ ions and exchangeable Ca^2+^ ions within the montmorillonite interlayers. The appearance of this band is also consistent with the XRD results, which showed the disappearance of the weak calcite reflection observed in RB (Figure 9). The absence of distinct carbonate reflections in the XRD pattern suggests that the carbonate-containing products formed during activation are predominantly amorphous or poorly crystalline. Consequently, their precise mineralogical composition cannot be unequivocally determined from the present data.

Collectively, the ATR–FTIR analysis (Figure 9) indicates that sodium activation does not significantly modify the montmorillonite framework but leads to the formation of carbonate-containing species. These species are associated with interlayer cation exchange and the transformation of calcium-bearing phases. These observations are consistent with the XRD results and the alterations in exchangeable cation composition observed following activation.

#### 3.2.3. Thermal Analysis of AB and Comparison with RB

**Figure 10:**
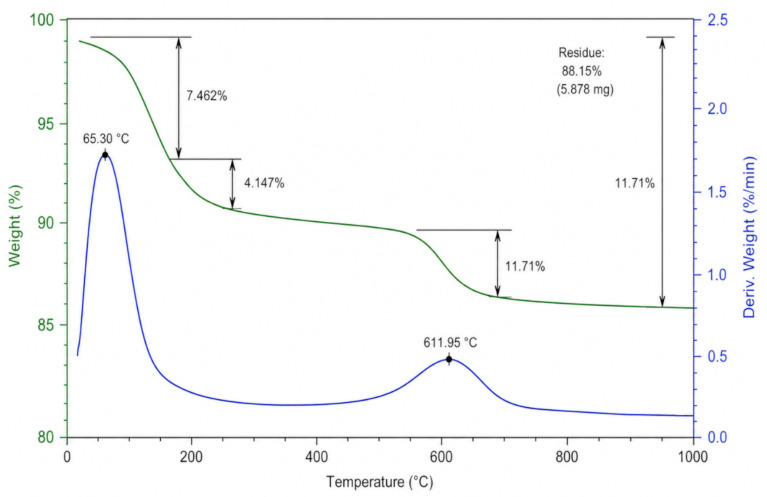
DCS/TG curves of AB sample.

**Figure 11:**
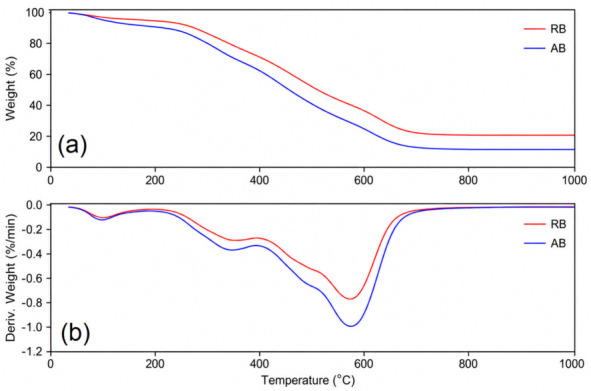
Comparison of (**a**) TG and (**b**) DSC curves of RB and AB samples.

The DCS/TG curves of sample AB are shown in Figure 10. For comparison, the combined DCS and TG curves of the RB and AB samples are provided in Figure 11, allowing for direct visualization of the thermal changes induced by sodium activation.

The thermal behavior of AB (Figure 10) closely resembled that of the raw bentonite (Figure 11), indicating that sodium activation did not substantially alter the thermal stability of the montmorillonite structure. The TG curve (Figure 10) exhibited a pronounced mass-loss effect below 200 °C, corresponding to the removal of physically adsorbed and interlayer water. This behavior is characteristic of smectite-group clay minerals and reflects the presence of hydrated exchangeable cations within the interlayer region.

A second thermal effect, characterized by a DSC maximum at approximately 611.95 °C (Figure 10), was attributed to the dehydroxylation of the montmorillonite structure. The preservation of this characteristic dehydroxylation process indicates that the fundamental layered aluminosilicate framework remained intact after activation. The slightly lower dehydroxylation temperature compared with RB (636 °C) (Figure 11b) may reflect modifications of the interlayer environment associated with the replacement of exchangeable Ca^2+^ ions by Na^+^ ions.

The total mass loss recorded up to 1000 °C was 11.71% (Figure 10), which is slightly lower than that observed for RB (13.77%) (Figure 11a). This result indicates that sodium activation did not promote additional thermal decomposition processes and suggests that the structural integrity of the bentonite was preserved during treatment. No significant additional thermal effects were observed throughout the investigated temperature range, indicating the absence of substantial quantities of thermally unstable secondary phases.

Although AB exhibited substantially higher free swelling and water absorption capacities than RB, its total TG mass loss was slightly lower (11.71% versus 13.77%). This apparent discrepancy arises because the swelling and water-absorption tests evaluate the maximum hydration capacity of the bentonite under excess-water conditions, whereas TG analysis reflects the amount of water retained in the dried material prior to heating. Therefore, the lower total mass loss of AB does not contradict its enhanced hydration behavior. Instead, the increased swelling capacity is primarily associated with Na^+^-induced expansion of the diffuse double layer and improved interlayer hydration rather than with a greater quantity of water retained in the equilibrated solid.

The comparison of the RB and AB samples (Figure 11a,b) shows that both samples exhibit very similar thermal behavior, indicating preservation of the montmorillonite structure following sodium activation. In both materials, the principal mass loss occurs below approximately 200 °C and is associated with the removal of physically adsorbed and interlayer water. The activated bentonite exhibits a slightly more pronounced low-temperature dehydration effect, consistent with its enhanced hydration capacity resulting from Na^+^ enrichment of the interlayer space. In contrast, the dehydroxylation process remains within the characteristic temperature range of montmorillonite, demonstrating that the layered aluminosilicate framework was not substantially altered during activation. The similarity of the thermal profiles of RB and AB, together with the preservation of the characteristic montmorillonite dehydroxylation behavior, is consistent with the XRD (Figure 1 and Figure 6) and ATR–FTIR results (Figure 2 and Figure 8), confirming that sodium activation primarily modified the interlayer chemistry while preserving the fundamental montmorillonite framework.

#### 3.2.4. Surface Characteristics of AB and Comparison with RB

**Figure 12:**
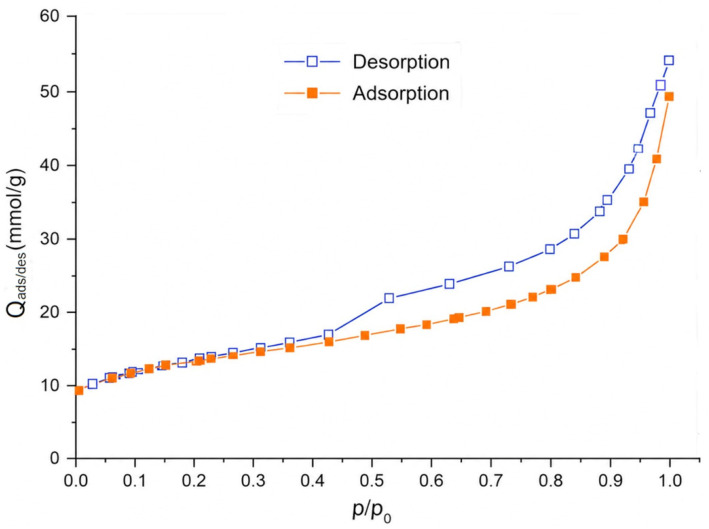
Nitrogen adsorption–desorption isotherm of AB sample.

**Figure 13:**
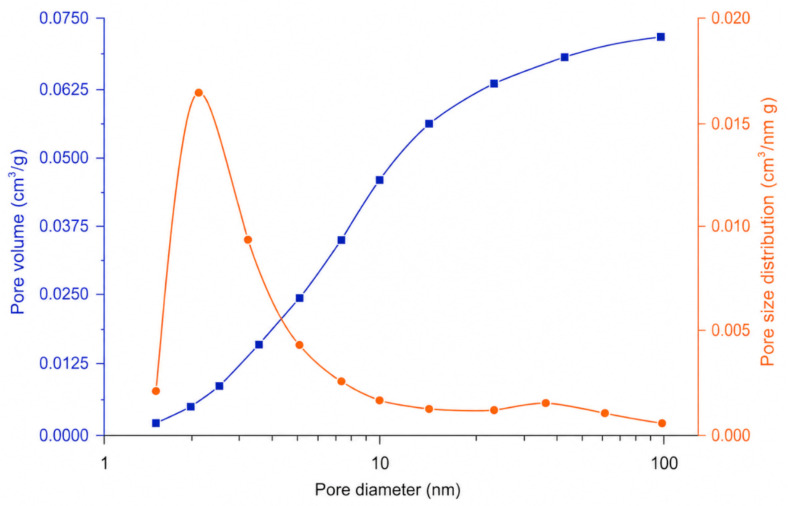
BJH pore size distribution and cumulative pore volume of AB sample.

**Figure 14:**
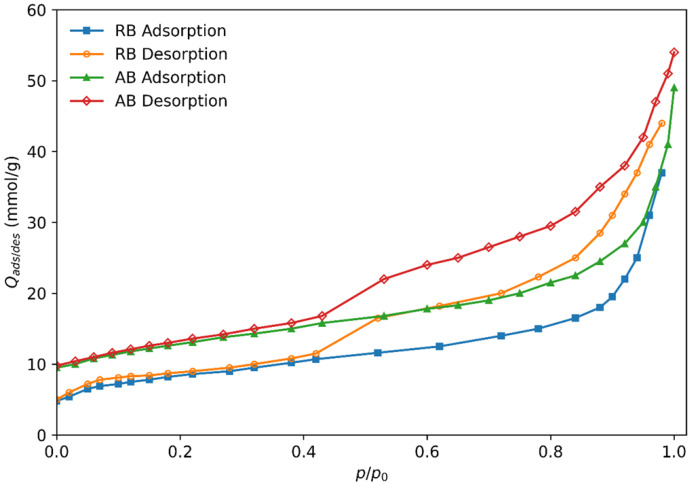
Comparison of nitrogen adsorption–desorption isotherms for AB and RB samples.

**Figure 15:**
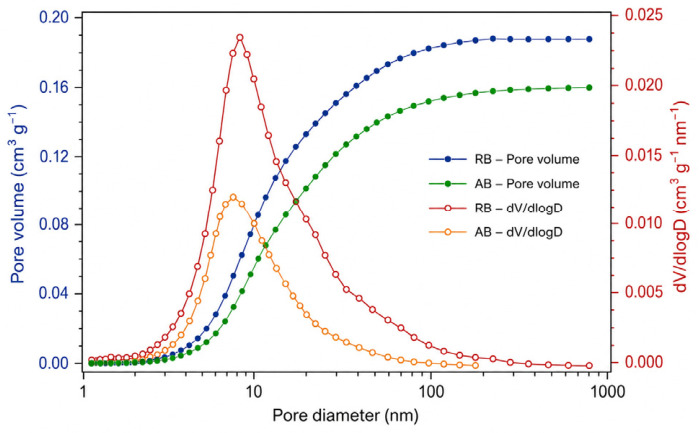
Comparison of BJH pore size distribution and cumulative pore volume of AB and RB samples.

Nitrogen adsorption–desorption isotherms and BJH pore size distribution curves for the sample AB are presented in Figure 12 and Figure 13, respectively. To facilitate direct comparison of the effects of sodium activation, the nitrogen adsorption–desorption isotherms of RB and AB are presented in one coordinate system in Figure 14, while the corresponding comparison of BJH pore size distribution and cumulative pore volume curves od AB and RB are provided in Figure 15. Finaly, the corresponding textural parameters are summarized in Table 8 together with those of the raw bentonite sample.

Direct comparison of the adsorption–desorption isotherms of RB and AB reveals a noticeable increase in nitrogen uptake after activation. As seen in Figure 12, both samples exhibit a type IV isotherm with a hysteresis loop characteristic of mesoporous materials, indicating that the overall pore structure was preserved during sodium activation. However, the higher adsorption volume observed for AB (Figure 14) throughout the relative pressure range suggests an increase in accessible surface area and pore volume. Similarly, cumulative pore volume of AB sample (Figure 13) and BJH pore size distribution curves (Figure 15) show comparable pore-size characteristics for both samples, indicating that activation primarily affected pore accessibility rather than the fundamental pore architecture.

As evidenced in Table 8, compared with RB, AB exhibited a 67.2% increase in specific surface area (SBET), accompanied by increases in total pore volume (26.2%), mesopore volume (25.8%), and micropore volume (221.7%). In contrast, the average pore diameter decreased slightly by 9.4%, whereas the modal pore diameter remained unchanged at approximately 3.7 nm.

The increase in surface area and pore volume is consistent with the replacement of exchangeable Ca^2+^ ions by Na^+^ ions, which promotes interlayer hydration, swelling, and improved dispersion of clay aggregates. As a result, a larger proportion of the internal surface becomes accessible for adsorption and ion-exchange processes. Despite these quantitative changes, the pore system remained predominantly mesoporous, with mesopores accounting for approximately 96% of the total pore volume in AB (Vmeso = 0.0771 cm^3^/g). The unchanged modal pore diameter further indicates that sodium activation enhanced pore accessibility without substantially altering the characteristic mesoporous architecture of the bentonite.

These textural modifications are consistent with the increases in cation exchange capacity, free swelling capacity, water absorption, and Atterberg consistency limits observed after activation. Collectively, the results demonstrate that sodium activation significantly improved the accessibility of adsorption and ion-exchange sites while preserving the fundamental pore structure of the montmorillonite-rich bentonite. Such modifications are expected to contribute positively to its performance as a binder in green sand foundry applications.

#### 3.2.5. Evaluation of the AB Sample for Foundry Applications

To evaluate the suitability of sample AB as a binder for green sand molding mixtures, its key physicochemical characteristics and bonding properties were determined (Table 9).

The activated bentonite exhibited favorable physicochemical properties for foundry use, including a free swelling capacity of 20 cm^3^, MB adsorption of 286.3 mg/g, and a cation exchange capacity (CEC) of 89.5 meq/100 g. The alkaline pH value (9.8) is consistent with sodium activation, while the refractoriness value of 1280 °C (SK 8) indicates sufficient refractoriness for conventional ferrous and non-ferrous casting applications.

The bonding tests demonstrated good performance of the activated bentonite in green sand mixtures containing 7 wt.% bentonite and 4 wt.% water. Green compressive strength, green shear strength, and wet tensile strength values of 60, 12, and 2.6 kPa, respectively, indicate effective bonding of sand grains and satisfactory mold integrity. Furthermore, the thermal stability value of 59 kPa after heating at 500 °C for 1 h demonstrates good retention of bonding capacity under elevated-temperature conditions. The measured gas permeability (100 cm^3^ min^−1^ cm^−2^) indicates sufficient permeability for efficient gas evacuation during casting, thereby reducing the likelihood of gas-related casting defects.

The favorable bonding performance of sample AB can be attributed to the physicochemical changes induced by sodium activation. The increased free swelling capacity, higher cation exchange capacity, and enlarged specific surface area enhance hydration, promote the formation of a stronger clay–water network, and improve the interaction between bentonite particles and sand grains. These improvements are consistent with the sodium-induced modification of the montmorillonite interlayer structure demonstrated by the XRD, ATR–FTIR, cation-exchange, and textural analyses presented in Section 3.2.1, Section 3.2.2, Section 3.2.3 and Section 3.2.4. Collectively, these effects contribute directly to the development of green strength and thermal stability in the molding mixture.

To assess the industrial relevance of the activated bentonite, selected properties were compared with values reported for commercial sodium bentonites used in green sand molding systems (Table 10).

The comparison presented in Table 10 demonstrates that the key technological properties of sample AB are comparable to those of commercial sodium bentonites used in green sand molding systems. The free swelling capacity of AB (20 cm^3^) falls within the typical range reported for commercial foundry-grade sodium bentonites (18–25 cm^3^), indicating adequate hydration and expansion behavior required for effective bond formation within the molding mixture. The CEC value (89.5 meq/100 g) is likewise within the range commonly reported for sodium bentonites (70–100 meq/100 g), confirming the successful conversion of the bentonite to a sodium-dominated form and its high capacity for water adsorption and ion exchange.

The measured green compressive strength (60 kPa) and wet tensile strength (2.6 kPa) are also comparable to values reported for commercial foundry bentonites, demonstrating sufficient bonding efficiency and mold integrity. In particular, wet tensile strength is an important indicator of resistance to cracking and erosion in the condensation zone during casting. The agreement of these parameters with industrial benchmarks confirms that the alkali activation treatment produced a bentonite with technological performance suitable for green sand molding applications.

Collectively, the favorable swelling behavior, high cation exchange capacity, enhanced surface characteristics, satisfactory bonding strength, adequate thermal stability, and sufficient gas permeability demonstrate that sodium activation successfully transformed the investigated Ca-rich bentonite into a material suitable for green sand foundry applications. The obtained properties are comparable to those of commercial sodium bentonites and indicate that the activated bentonite can serve as an effective binder for green sand molding mixtures used in both ferrous and non-ferrous casting operations.

#### 3.2.6. Integrated Discussion of Alkali Activation Effects

The combined results of the chemical, mineralogical, spectroscopic, thermal, textural, and technological analyses demonstrate that sodium activation substantially modified the physicochemical properties of the investigated bentonite while preserving the fundamental montmorillonite framework. Chemical analysis and exchangeable cation measurements revealed a pronounced increase in Na^+^ content accompanied by a decrease in Ca^2+^ content, confirming the successful conversion of the original Ca-rich bentonite into a sodium-dominated form.

*(1) Structural and mineralogical changes (as seen in Figure 1, Figure 6 and Figure 7):* The structural modifications induced by activation were evidenced by the shift in the montmorillonite (001) reflection in the XRD pattern and by the appearance of carbonate-related absorption bands in the ATR–FTIR spectrum. At the same time, the persistence of the characteristic montmorillonite reflections and hydroxyl-related infrared bands indicates that the layered aluminosilicate structure remained largely intact throughout the activation process. This conclusion is further supported by the DSC/TG results, which showed preservation of the characteristic dehydroxylation behavior of montmorillonite.

*(2) Spectroscopic and thermal evidence of interlayer modification:* ATR–FTIR spectroscopy (Figure 2 and Figure 8) confirms that sodium activation primarily modified the interlayer environment of montmorillonite without causing structural degradation. Increased intensity of water-related absorption bands and the presence of carbonate vibrations in AB indicate enhanced interlayer hydration and successful Na_2_CO_3_ treatment. Thermal analysis (Figure 3, Figure 10 and Figure 11) further supports this mechanism. The slightly increased low-temperature mass loss observed for AB reflects enhanced water uptake associated with sodium-enriched interlayers, whereas the unchanged dehydroxylation behavior confirms preservation of the montmorillonite framework. Together with XRD and ATR–FTIR data, the DSC/TG results demonstrate that the improved swelling, cation exchange capacity, and foundry performance originate from interlayer cation exchange and enhanced hydration rather than structural alteration.

*(3) Textural development and surface properties (as seen in Figure 12, Figure 13, Figure 14 and Figure 15):* Sodium activation also produced significant changes in the textural and physicochemical properties of the bentonite. Increases in specific surface area, pore volume, methylene blue adsorption, and cation exchange capacity indicate improved accessibility of adsorption and ion-exchange sites. These modifications were accompanied by substantial increases in free swelling capacity, water absorption, liquid limit, and plasticity index, reflecting enhanced hydration and diffuse double-layer expansion due to the predominance of exchangeable Na^+^ ions. Adsorption–desorption measurements provide further evidence of activation-induced textural evolution. The higher nitrogen uptake and increased BET surface area observed for AB indicate improved accessibility of adsorption sites and greater development of the pore structure. These changes are consistent with sodium-induced interlayer expansion and enhanced hydration, which collectively contributed to improved CEC, water absorption, and swelling capacity. The BET results establish a clear link between microscopic structural modification and macroscopic technological performance.

*(4) Technological performance enhancement:* Compared with raw bentonite, the alkali-activated bentonite exhibited marked improvements in all key properties relevant to foundry applications. The free swelling capacity increased from 7 to 20 cm^3^ (+185.7%), while water absorption increased from 116.50% to 280.78%. Similarly, the liquid limit and plasticity index increased from 118.7% to 244.6% and from 79.7% to 193.4%, respectively, demonstrating a substantially enhanced capacity for water retention and plastic deformation. These improvements are directly related to the replacement of exchangeable Ca^2+^ ions by Na^+^ ions within the montmorillonite interlayer space. Owing to their lower charge density, Na^+^ ions promote expansion of the diffuse double layer and facilitate water penetration into the interlayer region, resulting in enhanced hydration, swelling, and dispersion of clay particles.

*(5) Cation exchange and surface accessibility:* The effectiveness of sodium activation is further demonstrated by changes in exchangeable cation composition. Na^+^ content increased from 14.88 to 65.50 meq/100 g, while Ca^2+^ content decreased from 43.04 to 16.47 meq/100 g. Consequently, methylene blue adsorption increased from 238.5 to 286.3 mg/g and CEC increased from 74.6 to 89.5 meq/100 g. In parallel, BET surface area increased by approximately 67% (27.4 to 45.8 m^2^/g), total pore volume increased from 0.0638 to 0.0805 cm^3^/g, and micropore volume more than tripled (0.0023 to 0.0074 cm^3^/g), indicating enhanced accessibility of internal surfaces following activation.

*(6) Summary of physicochemical and technological changes:* The principal improvements induced by sodium activation are summarized in Table 11.

The data clearly demonstrate that sodium activation significantly enhances hydration, adsorption, ion-exchange, and textural properties. The most pronounced improvements are observed for free swelling capacity, micropore volume, and exchangeable sodium content, all directly associated with the transformation of the interlayer environment from Ca-dominated to Na-dominated.

*(7) Overall implications:* Na_2_CO_3_ activation effectively transformed the investigated Ca-rich bentonite into a sodium-dominated form with enhanced physicochemical and technological performance. The resulting material exhibits properties comparable to commercial sodium bentonites used in green sand molding systems, making it a promising binder for foundry applications.

The principal contribution of this work is not the sodium activation procedure itself, which is well established, but the comprehensive demonstration of how activation-induced changes in exchangeable cation composition, interlayer structure, and pore characteristics collectively govern the technological performance of the bentonite in foundry applications.

## 4. Conclusions

The present study demonstrated that sodium activation of a locally sourced calcium bentonite using 5 wt.% Na_2_CO_3_ effectively enhanced its physicochemical, textural, and technological properties, producing a sodium-dominated bentonite suitable for foundry applications. The optimum Na_2_CO_3_ dosage was identified on the basis of free swelling capacity, which increased from 7 to 20 cm^3^ after activation.

Sodium activation resulted in substantial improvements in the hydration and ion-exchange characteristics of the bentonite. The cation exchange capacity increased from 74.60 to 89.52 meq/100 g, methylene blue adsorption increased from 238.5 to 286.3 mg/g, and the BET specific surface area increased from 27.4 to 45.8 m^2^/g. These changes were accompanied by significant increases in water absorption capacity, liquid limit, and plasticity index, reflecting enhanced hydration, diffuse double-layer expansion, and accessibility of active adsorption sites following replacement of exchangeable Ca^2+^ ions by Na^+^ ions.

XRD, ATR–FTIR, and thermal analyses demonstrated that sodium activation primarily modified the interlayer chemistry while preserving the fundamental montmorillonite framework. The shift in the basal montmorillonite reflection from 1.47 to 1.23 nm, the increase in exchangeable Na^+^ content, the appearance of carbonate-related FTIR bands, and the preservation of the characteristic dehydroxylation behavior collectively confirmed successful sodium activation without significant structural degradation of the clay mineral.

The textural modifications induced by activation included increases in total pore volume and micropore volume while maintaining the predominantly mesoporous character of the bentonite. These changes contributed to improved accessibility of adsorption and ion-exchange sites and are consistent with the observed enhancements in swelling behavior and technological performance.

Comparison of the RB and AB characterization results demonstrates that sodium activation significantly modified the exchangeable cation composition, swelling behavior, and foundry-related properties of the investigated bentonite. The activated bentonite exhibited favorable foundry-related properties, including adequate refractoriness, high methylene blue adsorption capacity, satisfactory gas permeability, and improved bonding characteristics. The measured green compressive strength, wet tensile strength, free swelling capacity, and cation exchange capacity were comparable to values reported for commercial sodium bentonites used in green sand molding systems. These results confirm that the activated material satisfies the principal technological requirements for use as a binder in molds and cores for both ferrous and non-ferrous castings.

Overall, the study demonstrates that Na_2_CO_3_ activation is an effective and economically attractive route for upgrading locally available calcium bentonite into a value-added sodium bentonite. The observed relationships between interlayer cation exchange, structural and textural modifications, and foundry-relevant properties provide a useful basis for the development and utilization of locally sourced bentonite resources as alternatives to imported commercial sodium bentonites.

## Figures and Tables

**Table 1:** MB adsorption, exchangeable cations, and CEC of the RB sample.

MB Adsorption (mg/g)	CEC (meq/100 g)	Ca^2+^	Mg^2+^	Na^+^	K^+^	ΣCEC *
238.50	74.60	43.04	2.68	14.88	15.59	76.19

* Determined by the ammonium acetate method.

**Table 2:** Chemical composition of the RB sample.

Oxide	SiO_2_	Al_2_O_3_	CaO	MgO	Na_2_O	K_2_O	TiO_2_	Fe_2_O_3_	SO_3_	P_2_O_5_	LOI
(wt.%)	70.42	12.53	2.47	1.74	0.51	0.67	1.15	1.75	0.18	0.004	8.45

**Table 3:** Assignment of the principal ATR–FTIR absorption bands of the RB sample.

Wavenumber (cm^−1^)	Assignment	Ref.
~3620	Structural O–H stretching vibration (Al–Al–OH)	[20,24]
~1635	H–O–H bending vibration of adsorbed/interlayer water	[20,24]
~1030	Si–O stretching vibration of tetrahedral silicate sheets	[20,24]
~915	Al–Al–OH bending vibration	[20]
~792	Quartz/cristobalite Si–O vibration	[24,25]
~772	Quartz/cristobalite Si–O vibration	[24,25]
~520	Si–O–Al and Si–O–Si bending vibrations	[20]

**Table 4:** Textural parameters of RB sample.

Sample	SBET * (m^2^/g)	Vtotal ** (cm^3^/g)	Vmeso *** (cm^3^/g)	Vmicro ^+^ (cm^3^/g)	Davg ^++^ (nm)	Dmode ^+++^ (nm)
RB	27.4 ± 0.6	0.0638 ± 0.0012	0.0613 ± 0.0011	0.0023 ± 0.0002	8.5 ± 0.2	3.7 ± 0.1

* SBET—BET specific surface area; ** Vtotal—total pore volume; *** Vmeso—mesopore volume; ^+^ Vmicro—micropore volume; ^++^ Davg—average pore diameter; ^+++^ Dmode—modal pore diameter determined from the maximum of the BJH pore size distribution curve.

**Table 5:** Chemical composition of AB sample and its comparison with sample RB.

Oxide (wt.%)	SiO_2_	Al_2_O_3_	CaO	MgO	Na_2_O	K_2_O	TiO_2_	Fe_2_O_3_	SO_3_	P_2_O_5_	LOI
RB	70.42	12.53	2.47	1.74	0.51	0.67	1.15	1.75	0.18	0.004	8.45
AB	69.30	11.09	1.85	1.68	2.22	0.50	0.95	1.25	0.12	0.002	10.87
Change (%)	−1.59	−11.49	−25.10	−3.4	335.29	−25.37	−17.39	−28.57	−33.33	−50.0	28.64

**Table 6:** Free swelling, Atterberg consistency limits, and water absorption capacities of RB and AB samples.

Sample	Free Swelling (cm^3^)	WLL * (%)	WPL ** (%)	PI *** (%)	Water Absorption (%)
RB	7.0 ± 0.3	118.70	39.03	79.67	116.50
AB	20.0 ± 0.5	244.60	51.20	193.40	280.78

* WLL—liquid limit; ** WPL—plastic limit; and *** PI—plasticity index.

**Table 7:** Exchangeable cations and cation exchange capacities of RB and AB samples.

Sample	Ca^2+^	Mg^2+^	Na^+^	K^+^	Σ (meq/100 g)	MB Absorption (mg/g)	CEC (meq/100 g)
RB	43.04	2.68	14.88	15.59	76.19	238.50	74.60
AB	16.47	10.88	65.50	2.74	95.24	286.28	89.52

**Table 8:** Surface characteristics of AB sample in comparison with RB sample.

Sample	SBET (m^2^/g)	Vtotal (cm^3^/g)	Vmeso (cm^3^/g)	Vmicro (cm^3^/g)	Davg (nm)	Dmode (nm)
RB	27.4 ± 0.6	0.0638 ± 0.0012	0.0613 ± 0.0011	0.0023 ± 0.0002	8.5 ± 0.2	3.7 ± 0.1
AB	45.8 ± 0.9	0.0805 ± 0.0015	0.0771 ± 0.0014	0.0074 ± 0.0003	7.7 ± 0.2	3.7 ± 0.1
Change (%)	67.15	26.18	25.78	221.74	−9.41	0.00

**Table 9:** Quality parameters and bonding properties of the AB sample relevant to foundry applications.

Physical and Chemical Parameters	
Moisture content	10.8 wt.%
Fineness	100% passing 0.074 mm
pH	9.8
Free swelling	20.0 ± 0.5 cm^3^
Refractoriness (softening point)	1280 °C (SK 8)
MB absorption	286.3 ± 3.2 mg/g
CEC	89.5 ± 1.4 meq/100 g
Bonding Properties *	
Green compressive strength	60 ± 2 kPa
Green shear strength	12 ± 1 kPa
Wet tensile strength	2.6 ± 0.1 kPa
Thermal stability (500 °C, 1 h)	59 ± 2 kPa
Gas permeability	100 ± 4 cm^3^/min·cm^2^

* Test mixture: 7 wt.% bentonite, 4 wt.% water.

**Table 10:** Comparison of selected properties of sample AB with commercial sodium bentonites reported in the literature and technical specifications.

Property	AB	Commercial Sodium Bentonite	Ref.
Free swelling (cm^3^)	20	18–25	[50,52]
CEC (meq/100 g)	89.5	70–100	[29,50]
Green compressive strength (kPa)	60	50–80	[26,53]
Wet tensile strength (kPa)	2.6	2–5	[52,54]

**Table 11:** Summary of physicochemical, textural, and technological changes induced by sodium activation.

Property	RB	AB	Relative Change (%)
Free swelling (cm^3^)	7	20	+185.7
Exchangeable Na^+^ (meq/100 g)	14.88	65.50	+340.2
Exchangeable Ca^2+^ (meq/100 g)	43.04	16.47	−61.7
MB adsorption (mg/g)	238.50	286.28	+20.0
CEC (meq/100 g)	74.60	89.52	+20.0
BET surface area (m^2^/g)	27.4	45.8	+67.2
Total pore volume (cm^3^/g)	0.0638	0.0805	+26.2
Micropore volume (cm^3^/g)	0.0023	0.0074	+221.7
Water absorption (%)	116.50	280.78	+141.0
Liquid limit (%)	118.70	244.60	+106.1
Plasticity index (%)	79.67	193.40	+142.7

## Data Availability

The original contributions presented in this study are included in the article. Further inquiries can be directed to the corresponding author.

## Abbreviations

ATR	Attenuated Total Reflectance
BET	Brunauer–Emmett–Teller
BJH	Barrett–Joyner–Halenda
CEC	Cation Exchange Capacity
LOI	Loss on Ignition
MB	Methylene Blue
PCE	Pyrometric Cone Equivalent
PI	Plasticity Index
SBET	Specific Surface Area Determined by the BET Method
Vtotal	Total Pore Volume
Vmeso	Mesopore Volume
Vmicro	Micropore Volume
WLL	Liquid Limit
WPL	Plastic Limit
Davg	Average Pore Diameter
Dmode	Modal Pore Diameter from Pore Size Distribution
2θ	Diffraction Angle in X-ray Diffraction

## References

[1] Forano C., Hibino T., Leroux F., Taviot-Guého C., Bergaya F., Lagaly G. (2006). Layered double hydroxides. Developments in Clay Science.

[2] Kadir S., Külah T., Erkoyun H., Uyanık N.Ö., Eren M., Elliott W.C. (2021). Mineralogy, geochemistry, and genesis of bentonites in Upper Cretaceous pyroclastics of the Bereketli Member of the Reşadiye Formation, Reşadiye (Tokat), Turkey. Appl. Clay Sci..

[3] Lee Y., Kim P., Kim H., Seoung D. (2020). Comparative compressibility of smectite group under anhydrous and hydrous environments. Materials.

[4] Zhou W., Wang F. (2025). Sorption of Se(IV) on Gaomiaozi bentonite: Batch and spectroscopic studies. J. Environ. Radioact..

[5] Noh D.-H., Kim S., Calaunan J.M.F.V., Feng Y., Eun J., Kim Y.-R. (2025). Bentonite as an engineered barrier material for nuclear waste repository: Geotechnical perspectives, key properties, knowledge gaps, and opportunities. Nucl. Eng. Technol..

[6] Jiang L., Wang H., Miao Y., Zhao Q., Min M., Qiu J., Pu H. (2025). Preparation and properties of cross-linked polymer/bentonite nanocomposite for containment of chemically aggressive liquids. J. Rock Mech. Geotech. Eng..

[7] Hussain F., Kamran M., Inam A., Ishtiaq M., Hassan M.H., Riaz F., Khan T., Ammar M., Salman M. (2025). Evaluation of foundry properties of locally available molding sand with bentonite clay additions for cost-effective aluminum alloy casting. Arch. Foundry Eng..

[8] Murray H.H. (2006). Bentonite applications. Developments in Clay Science.

[9] Boylu F. (2011). Optimization of foundry sand characteristics of soda-activated calcium bentonite. Appl. Clay Sci..

[10] Khan K., Khan S.A., Saleem M.U., Ashraf M. (2017). Improvement of locally available raw bentonite for use as drilling mud. Open Constr. Build. Technol. J..

[11] Ma Y., Li Q., Chen X., Zhang Y., Yang Y., Zhong Q. (2024). Reducing bentonite usage in iron ore pelletization through a novel polymer-type binder: Impact on pellet induration and metallurgical properties. J. Mater. Res. Technol..

[12] Terzić A., Pezo L., Mijatović N., Stojanović J., Kragović M., Miličić L., Andrić L. (2018). The effect of alternations in mineral additives (zeolite, bentonite, fly ash) on physico-chemical behavior of Portland cement based binders. Constr. Build. Mater..

[13] Hasan K., Jaya R.P., Yahaya F.M. (2024). Application of bentonite in cement-based composites: A review of current status, challenges and future prospects. J. Build. Eng..

[14] Ousaleh H.A., Sair S., Mansouri S., Abboud Y., Zahouily M., Faik A., Bouari A.E. (2022). Enhanced inorganic salts stability using bentonite clay for high-performance and low-cost thermochemical energy storage. J. Energy Storage.

[15] Che J., Bronchy M., Kannan A., Boissière C., Zakri C., Poulin P., Yuan J. (2025). Waterborne polymer composites with surface-charge-modulated bentonite nanosheets for high-energy storage. Chem. Eng. J..

[16] Chokri M., Azougagh O., El Bojaddayni I., Jalafi I., El Ouardi Y., Jilal I., Ahari M., Salhi A., El Idrissi A., Bendahhou A. (2025). Progress in bentonite clay modification and enhancing properties to industrial applications: A review. Mater. Chem. Phys..

[17] Shahzadi F., Sun X.-F., Sheraz M. (2025). Sustainable Cellulose–Bentonite composites for wastewater treatment. Materials.

[18] Sun Y., Li Y., Xu Y., Liang X., Wang L. (2015). In situ stabilization remediation of cadmium (Cd) and lead (Pb) co-contaminated paddy soil using bentonite. Appl. Clay Sci..

[19] Uddin F., Zoveidavianpoor M. (2018). Montmorillonite: An Introduction to Properties and Utilization. Current Topics in the Utilization of Clay in Industrial and Medical Applications.

[20] Krupskaya V., Zakusin S., Tyupina E., Dorzhieva O., Zhukhlistov A., Belousov P., Timofeeva M. (2017). Experimental Study of Montmorillonite Structure and Transformation of Its Properties under Treatment with Inorganic Acid Solutions. Minerals.

[21] Perelomov L., Gertsen M., Burachevskaya M., Hemalatha S., Vijayalakshmi A., Perelomova I., Atroshchenko Y. (2024). Organoclays based on bentonite and various types of surfactants as heavy metal remediants. Sustainability.

[22] Luckham P.F., Rossi S. (1999). The colloidal and rheological properties of bentonite suspensions. Adv. Colloid Interface Sci..

[23] Bajestani M.S., Nasir O., Oh W.T. (2023). Properties of Bentonite-Based Sealing Materials during Hydration. Minerals.

[24] Damian G., Damian F., Szakács Z., Iepure G., Aştefanei D. (2021). Mineralogical and Physico-Chemical Characterization of the Oraşu-Nou (Romania) Bentonite Resources. Minerals.

[25] Henderson J.H., Jackson M.L., Syers J.K., Clayton R.N., Rex R.W. (1971). Cristobalite authigenic origin in relation to Montmorillonite and quartz origin in bentonites. Clays Clay Miner..

[26] Khan M.M., Mahajani S.M., Jadhav G.N. (2021). Transformation of bentonite used in green sand molds during metal casting process and its relevance in sand reclamation. Appl. Clay Sci..

[27] Chang Y., Hocheng H. (2001). Flowability of bentonite-bonded green molding sand. J. Mater. Process. Technol..

[28] Bahranowski K., Klimek A., Gaweł A., Serwicka E.M. (2021). Rehydration driven Na-Activation of Bentonite—Evolution of the clay structure and composition. Materials.

[29] Macheca A.D., Mapossa A.B., Cumbane A.J., Sulemane A.E., Tichapondwa S.M. (2022). Development and characterization of NA2CO3-Activated Mozambican Bentonite: Prediction of optimal activation conditions using statistical design modeling. Minerals.

[30] Rosário J.A., Silva L.A., Moura G.B.G., Gusatti M., Lima R.B., Brys M.E.P., Kuhnen N.C., Riella H.G. (2010). Influence of Alkaline Activation over Swelling and Cation Exchange Capacity on Bentonites. Mater. Sci. Forum.

[31] Naka A., Flores G., Inui T., Sakanakura H., Katsumi T. (2019). Hydraulic performance and chemical compatibility of a powdered Na-bentonite geosynthetic clay liner permeated with mine drainage. Soilis Found..

[32] Jing T., Yuan S., Liu X., Liu Y., Xu H., Zhou W., Lin P., Jiang G. (2026). Effect of alkali activation on swelling suppression and microstructural development in Geopolymer-Stabilized bentonite. Polymers.

[33] Xu L., Li J., Ma M., Gao Z., Li W., Zhu Y., Lu G., Zhu Y. (2026). Iron and montmorillonite content effects on the thermal stability of casting-grade bentonite. Mater. Today Commun..

[34] Obradović M., Daković A., Smiljanić D., Marković M., Ožegović M., Krstić J., Vuković N., Milojević-Rakić M. (2023). Bentonite Modified with Surfactants—Efficient Adsorbents for the Removal of Non-Steroidal Anti-Inflammatory Drugs. Processes.

[35] Ratkievicius L.A., Da Cunha Filho F.J.V., De Barros Neto E.L., Santanna V.C. (2016). Modification of bentonite clay by a cationic surfactant to be used as a viscosity enhancer in vegetable-oil-based drilling fluid. Appl. Clay Sci..

[36] Bors J. (2000). Organophilic bentonites as adsorbents for radionuclides I. Adsorption of ionic fission products. Appl. Clay Sci..

[37] Capelezzo A.P., Celuppi L.C.M., Macuvele D.L.P., Zeferino R.C.F., Zanetti M., Bender J.P., De Mello J.M.M., Fiori M.A., Riella H.G. (2023). Obtaining and characterization of bentonite organophilic incorporated with geranyl acetate and its application as mycotoxins’ binder in simulated gastrointestinal fluids. Appl. Clay Sci..

[38] Ouellet-Plamondon C.M., Stasiak J., Al-Tabbaa A. (2014). The effect of cationic, non-ionic and amphiphilic surfactants on the intercalation of bentonite. Colloids Surf. A Physicochem. Eng. Asp..

[39] Andrunik M., Bajda T. (2019). Modification of Bentonite with Cationic and Nonionic Surfactants: Structural and Textural Features. Materials.

[40] Fu X.-L., Zhuang H., Reddy K.R., Jiang N.-J., Du Y.-J. (2023). Novel composite polymer-amended bentonite for environmental containment: Hydraulic conductivity, chemical compatibility, enhanced rheology and polymer stability. Constr. Build. Mater..

[41] Cui Q., Chen B. (2023). Review of polymer-amended bentonite: Categories, mechanism, modification processes and application in barriers for isolating contaminants. Appl. Clay Sci..

[42] Arabmofrad S., Lazzara G., Miller R., Jafari S.M. (2024). Surface modification of bentonite and montmorillonite as novel nano-adsorbents for the removal of phenols, heavy metals and drug residues. Adv. Colloid Interface Sci..

[43] Uddin M.K. (2016). A review on the adsorption of heavy metals by clay minerals, with special focus on the past decade. Chem. Eng. J..

[44] (2025). Standard Test Method for Swell Index of Clay Mineral Component of Geosynthetic Clay Liners.

[45] (2018). Standard Test Methods for Liquid Limit, Plastic Limit, and Plasticity Index of Soils.

[46] (2015). Geotechnical Investigation and Testing—Laboratory Testing of Soil—Part 2: Determination of Bulk Density.

[47] (2012). Soil, Testing Procedures and Testing Equipment—Determination of Water Absorption.

[48] (2022). Standard Test Method for Pyrometric Cone Equivalent (PCE) of Fireclay and High-Alumina Refractory Materials.

[49] (2020). Refractory Products—Determination of Pyrometric Cone Equivalent (Refractoriness).

[50] Hmeid H.A., Akodad M., Halim M.E., Omdi F.E., Baghour M., Skalli A., Sadik C., Gueddari H., Chahban M., Yousfi Y.E. (2025). Comparative analysis of Na^+^ and Ca^2+^ ion effects on the physical-chemical properties of Bentonite: Implications for industrial applications. Ore Energy Resour. Geol..

[51] Mooshaee M.R., Sabour M., Kamza E. (2022). The swelling performance of raw and modified bentonite of geosynthetic clay liner as the leachate barrier exposed to the synthetic E-waste leachate. Heliyon.

[52] Foundry Bentonite Specifications (Canbensan Foundry Grade Bentonite Technical Specifications). https://www.canbensan.com/products/foundry-grade-bentonite.

[53] Kamińska J., Stachowicz M., Puzio S., Angrecki M. (2022). Studies of mechanical and technological parameters and evaluation of the role of lustrous carbon carriers in green moulding sands with hybrid bentonite. Arch. Civ. Mech. Eng..

[54] Schiebel K., Jordan G., Kaestner A., Schillinger B., Boehnke S., Schmahl W.W. (2017). Neutron radiographic study of the effect of Heat-Driven water transport on the tensile strength of Bentonite-Bonded moulding sand. Transp. Porous Media.

